# LncRNA UCA1 Antagonizes Arsenic‐Induced Cell Cycle Arrest through Destabilizing EZH2 and Facilitating NFATc2 Expression

**DOI:** 10.1002/advs.201903630

**Published:** 2020-04-13

**Authors:** Zheng Dong, Ming Gao, Changying Li, Ming Xu, Sijin Liu

**Affiliations:** ^1^ State Key Laboratory of Environmental Chemistry and Ecotoxicology Research Center for Eco‐Environmental Sciences Chinese Academy of Sciences Beijing 100085 China; ^2^ College of Resources and Environment University of Chinese Academy of Sciences Beijing 100049 China; ^3^ Liver Research Center Beijing Friendship Hospital Capital Medical University Beijing 100050 China

**Keywords:** arsenic, cell cycle arrest, enhancer of zeste homolog 2 (EZH2), lncRNA, tumorigenesis, urothelial cancer associated 1 (UCA1)

## Abstract

Arsenic (As) is a widespread metalloid contaminant, and its internal exposure is demonstrated to cause serious detrimental health problems. Albeit considerable studies are performed to interrogate the molecular mechanisms responsible for As‐induced toxicities, the exact mechanisms are not fully understood yet, especially at the epigenetic regulation level. In the present study, it is identified that long non‐coding RNA (lncRNA) urothelial cancer associated 1 (UCA1) alleviates As‐induced G2/M phase arrest in human liver cells. Intensive mechanistic investigations illustrate that UCA1 interacts with enhancer of zeste homolog 2 (EZH2) and accelerates the latter's protein turnover rate under normal and As‐exposure conditions. The phosphorylation of EZH2 at the Thr‐487 site by cyclin dependent kinase 1 (CDK1) is responsible for As‐induced EZH2 protein degradation, and UCA1 enhances this process through increasing the interaction between CDK1 and EZH2. As a consequence, the cell cycle regulator nuclear factor of activated T cells 2 (NFATc2), a downstream target of EZH2, is upregulated to resist As‐blocked cell cycle progress and cytotoxicity. In conclusion, the findings decipher a novel prosurvival signaling pathway underlying As toxicity from the perspective of epigenetic regulation: UCA1 facilitates the ubiquitination of EZH2 to upregulate NFATc2 and further antagonizes As‐induced cell cycle arrest.

## Graphical abstract

Long non‐coding RNA UCA1 alleviates As‐induced cell cycle arrest in HepG2 hepatocytes. UCA1 interacts with EZH2 protein to promote its prosurvival function. UCA1 facilitates the interaction between kinase CDK1 and EZH2 to enhance the latter's phosphorylation at the Thr‐487 site. NFATc2 is a final executor of UCA1/EZH2 signaling to antagonize the As‐blocked cell cycle.

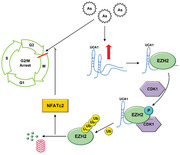

## 1 Introduction

Arsenic (As) is a ubiquitous toxic metalloid with multiple chemical forms, gives rise to a wide spectrum of health problems and receives particular attention.^[^ ^1^, ^2^, ^3^ ^]^ As exposure has been documented to cause plenty of carcinogenic and noncarcinogenic adverse biological effects, including hepatotoxicity, embryotoxicity, nephrotoxicity, and neurovirulence.^[^ ^4^, ^5^, ^6^ ^]^ At the cellular level, As could trigger various cytotoxicity through oxidative stress, metabolic disorders, apoptosis, and so on.^[^ ^7^, ^8^ ^]^ The genetic toxicity of As has been demonstrated in mounting in vitro and in vivo research, which affects genomic stability via DNA damage, chromosomal aberrations or impaired DNA repair.^[^ ^9^ ^]^ Besides, As‐induced epigenetic modifications have also been shown to play a vital role in altering gene regulation.^[^ ^10^, ^11^, ^12^, ^13^ ^]^ Although the genetic alteration and epigenetic dysregulation have been documented to be important mechanisms involved in the As‐induced toxic events, the underlying molecular bases remain elusive. Of note, the intricate defense regulatory networks against the stress responding to toxic pollutants still need further research.

The molecular mechanisms of epigenetics include DNA methylation, histone modification, and noncoding RNAs expression.^[^ ^14^, ^15^ ^]^ To date, growing studies have revealed that diversified environmental contaminant contribute to epigenetic modification, such as particulate matters (PM), heavy metals, persistent organic pollutants (POPs), and so on.^[^ ^16^, ^17^, ^18^ ^]^ The exposure of pollutants could modify the function and stability of the genome, and promote the development and progression of pollutant‐related disorders, especially the malignant tumors.^[^ ^16^, ^19^, ^20^ ^]^ To this end, it would be illuminating to probe into the concrete mechanisms of contaminant‐induced epigenetic alterations. As a renowned epigenetic regulatory molecule, enhancer of zeste homolog 2 (EZH2) is well known as methyltransferase for the tri‐methylation of lysine 27 on histone H3 (H3K27me3),^[^ ^21^ ^]^ and further results in gene silencing and exhibits oncogenic function.^[^ ^22^, ^23^ ^]^ Till now, the biological functions of EZH2 in pollutant‐induced toxic effects are almost unexploited. Aberrant expression and function of EZH2 had been detected in the utero and mammary tissues of mice exposed to bisphenol‐A.^[^ ^24^ ^]^ Furthermore, PM2.5 had been demonstrated to regulate the expression of EZH2 to further induce G2/M phase arrest in the A549 pneumonocytes.^[^ ^25^ ^]^ Nevertheless, whether there are other more molecules participate in the As‐mediated epigenetic alteration has not been clearly elucidated, and particularly the epigenetic regulating effect of EZH2 is worth intensive study.

Long non‐coding RNAs (lncRNAs) are the transcripts longer than 200 nucleotides without a protein‐coding function,^[^ ^26^ ^]^ and have been verified to regulate gene expression and protein functions to maintain cellular homeostasis.^[^ ^27^, ^28^, ^29^ ^]^ Recent evidence has suggested the necessary contribution of lncRNAs to environmental pollutant‐related toxicological responses and a large array of human disorders.^[^ ^30^, ^31^ ^]^ Our previous research has indicated that lncRNA urothelial cancer associated 1 (UCA1) played a protective role in preventing hepatocytes from As‐induced autophagy‐dependent cell death.^[^ ^32^ ^]^ In the present study, we aimed to elucidate the UCA1‐regulated EZH2‐initiated signaling pathways in HepG2 hepatocytes under As exposure. Here, our results unearthed a novel prosurvival molecular mechanism responding to As‐induced cell cycle arrest. Specifically, UCA1 interacted with EZH2 and regulated its phosphorylation and ubiquitylation through recruiting cyclin dependent kinase 1 (CDK1). The reduction of EZH2 further activated cell cycle‐related nuclear factor of activated T cells 2 (NFATc2) to antagonize cytotoxicity under the As stress. Thus, our study unveiled a new cell‐protective molecular signaling pathway underlying As‐induced cell cycle arrest.

## 2 Results

### 2.1 EZH2 Regulates As‐Induced Cell Cycle Arrest

**Figure 1:**
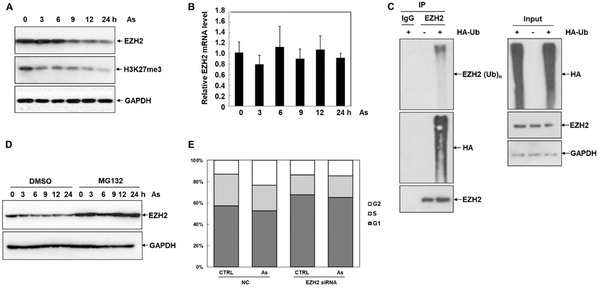
As induces cell G2/M phase arrest via regulating EZH2. A) Western blot analysis of the protein levels of EZH2 and H3K27me3 in HepG2 cells after treatment with 10 µmol As for 0, 3, 6, 9, 12, and 24 h (*n* = 3). B) Relative levels of EZH2 in HepG2 cells exposed to 10 µmol As at different time points were detected by qRT‐PCR analysis (*n* = 3). C) HepG2 cells transfected with HA‐ubiquitin were immunoprecipitated with anti‐EZH2 or IgG, and blotted with antibodies against EZH2, HA, and ubiquitin (*n* = 3). D) The protein concentration of EZH2 responding to 10 µmol As for 0–24 h in HepG2 cells pretreated with 1 µmol MG132 or DMSO (*n* = 3). E) Cell cycle distribution in scrambled control and EZH2 siRNA HepG2 cells in response to AS was analyzed via flow cytometry, after staining by PI (*n* = 3).

To investigate whether EZH2 was involved in As‐induced cytotoxicity of liver cells, we firstly deliberately detected the expression of EZH2 protein treated with As in HepG2 cells at different time points. As shown in **Figure** **1**A, the contents of EZH2 were diminished upon As stress for 3 h, and significantly attenuated for 9, 12, and 24 h, displaying that As could initiate EZH2 induction quickly. To further investigate whether As treatment obstructed the regulatory function of EZH2, we looked into the levels of H3K27me3. Similar to the decreasing tendency of EZH2, the protein contents of H3K27me3 in HepG2 cells were decreased with a time‐dependent manner upon treatment with As (Figure 1A). These observations uncovered that As treatment significantly decreased the protein levels of EZH2, and affected its biological function as the histone methyltransferase.

Next, we endeavored to unveil the underlying mechanisms for the reduction of EZH2 protein under As treatment. As shown in Figure 1B, quantitative reverse transcriptase‐PCR (qRT‐PCR) analyses illustrated that the mRNA levels of EZH2 were not markedly induced by As treatment, ruling out the regulation of As on the transcription or mRNA stability of EZH2. As a crucial post‐translational modification process, ubiquitination plays significant roles in regulating the stability and functions of proteins.^[^ ^33^, ^34^, ^35^ ^]^ Hence, we performed ubiquitination assays to assess the stability of the EZH2 protein under As stress. EZH2 protein was immunoprecipitated from HepG2 cells transfected with HA‐ubiquitin, and the results revealed that EZH2 could be ubiquitinated through attaching to the ubiquitin (Figure 1C). The levels of EZH2 were further determined in HepG2 cells incubated with the proteasome inhibitor MG132. As illustrated in Figure 1D, EZH2 was observably increased under MG132 treatment, compared to the untreated cells, indicating that As could promote the degradation of EZH2 protein through the ubiquitin–proteasome pathway. Together, our findings demonstrated that As could attenuate the stability of EZH2 through promoting its ubiquitination.

A large number of studies have shown that As could block regular cell cycle progression and induce cell apoptosis in vitro and in vivo.^[^ ^36^, ^37^, ^38^ ^]^ As illustrated in Figure 1E, the cell cycle distribution in HepG2 cells was analyzed by flow cytometry. Consistent with existing research, As treatment caused a significantly increased percentage of cells in the G2 phase, and companied with a reduction in the S phase, compared to the control groups. To further elucidate the regulation of As‐induced cell cycle arrest by EZH2, we performed the knockdown of EZH2 through RNA interference (RNAi). The cell cycle arrest was attenuated upon EZH2 knockdown regardless of As treatment, relative to the scrambled control cells (Figure 1E). Consistent with this finding, the regulation of EZH2 in As‐induced cell cycle arrest was determined in normal human kidney HK2 cells (Figure S1, Supporting Information). Therefore, these data suggested the crucial role of EZH2 reduction in antagonizing As toxicity.

### 2.2 LncRNA UCA1 Interacts with EZH2 to Regulate As‐Induced Cell Cycle Arrest

**Figure 2:**
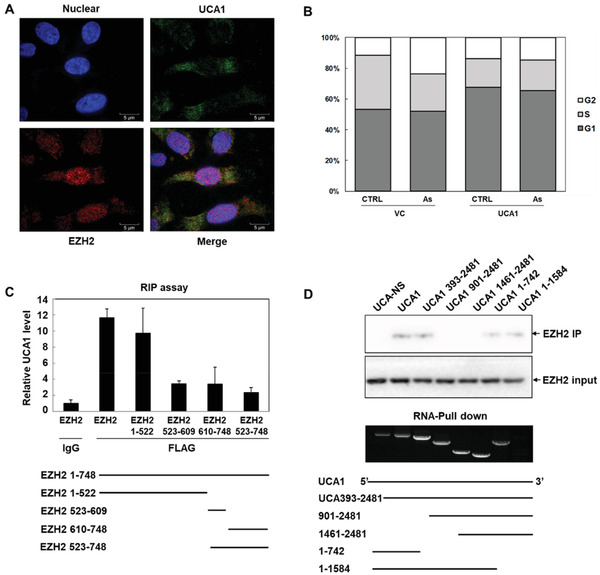
LncRNA UCA1 interacts with EZH2 on structural levels. A) The location of UCA1 (green) and EZH2 (red) in HepG2 cells was detected by FISH assays, and nuclei were stained by DAPI (blue) (*n* = 3). B) Flow cytometry analyses determined the cell cycle distribution in vehicle control and UCA1 overexpression HepG2 cells responding to As, after staining by PI (*n* = 3). C) The binding between UCA1 and EZH2 full length or truncations (amino acids 1–522, 523–609, 610–748, and 523–748) was detected via qRT‐PCR assay in RIP assay (*n* = 3). D) The binding of EZH2 to full‐length UCA1 and its truncations (393–2481, 1–742, and 1–1584) was analyzed by Western blot analysis in RNA‐pull down assay (*n* = 3).

Our previous study has revealed that UCA1 was remarkably induced by As treatment, which contributed to antagonizing As‐induced autophagic flux blockage.^[^ ^32^ ^]^ Additionally, recent studies have reported that UCA1 could interact with EZH2 to exert its epigenetic regulatory functions.^[^ ^39^ ^]^ Consequently, we focused on unveiling the interaction between UCA1 and As‐induced cell cycle arrest regulated by EZH2. Since the biological functions of lncRNAs and proteins depended on their subcellular localization,^[^ ^40^ ^]^ fluorescence in situ hybridization (FISH) assays were performed to demonstrate the distribution of UCA1 and EZH2 in HepG2 cells. As shown in **Figure** **2**A, EZH2 was mostly distributed in the nucleus, and UCA1 was expressed in both the nucleus and the cytoplasm, indicating that UCA1 might interact with EZH2 in the nucleus to exert potential biological functions. We then identified whether UCA1 involved in the regulation of As‐induced cell cycle arrest. As illustrated in Figure 2B, contrary to the regulatory manner of EZH2, As‐induced cell cycle arrest was ameliorated in the UCA1 overexpression cells.

To gain a more explicit sense of the binding between UCA1 and the EZH2 protein, we designed a series of truncation mutants of EZH2 and UCA1. As shown in Figure 2C, RNA‐binding protein immunoprecipitation (RIP) assays were performed to detect the regions of EZH2 bound to UCA1. The construct of EZH2, containing between amino acids 1 and 522, bound observably to UCA1, whereas the constructs containing residues 523–609, 610–748, and 523–748 presented weak bindings, demonstrating that UCA1 bound to the N terminus of EZH2. Besides, RNA‐Pull down assays were implemented to identify the binding region of UCA1 to EZH2 (Figure 2D). Western blot analyses showed that EZH2 proteins were enriched in the pulldown products of full‐length UCA1 and its truncations (393–2481, 1–742, and 1–1584), compared with nonsense RNA, while the truncations containing nucleotides 901–2481 and 1461–2481 did not precipitate any protein, suggesting that EZH2 might interact with the UCA1 between nucleotides 393 and 742. These data confirmed the interaction between EZH2 and UCA1 on structural levels, contributing to further elucidating the regulatory mechanisms of EZH2 by UCA1.

### 2.3 EZH2 Expression Is Regulated by LncRNA UCA1 in Response to As Stress

**Figure 3:**
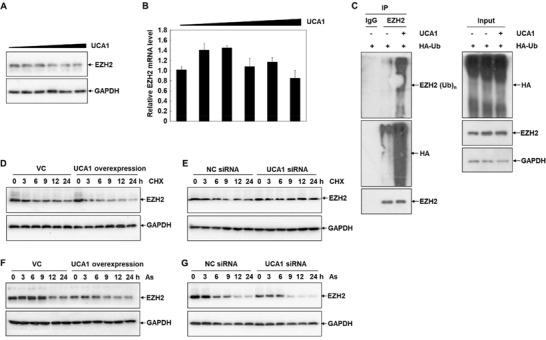
UCA1 regulates EZH2 expression upon As treatment. A,B) The protein levels and mRNA levels of EZH2 in HepG2 cells transfected with indicated concentrations of UCA1 overexpression plasmids were determined by Western blot analysis and qRT‐PCR assay (*n* = 3). C) HepG2 cells transfected with the plasmids of UCA1 and HA‐tagged ubiquitin were immunoprecipitated with anti‐EZH2 or IgG, and blotted with anti‐EZH2 antibody, anti‐HA antibody and anti‐ubiquitin antibody (*n* = 3). D,E) The protein contents of EZH2 in HepG2 cells transfected with UCA1 overexpression plasmids and UCA1 siRNA in response to 1 µmol CHX treatment for 0–24 h were analyzed by Western blot analysis (*n* = 3). F,G) Western blot analysis of the expression of EZH2 in HepG2 cells transfected with UCA1 overexpression plasmids and UCA1 siRNA exposed to 10 µmol As at different time points (*n* = 3).

Thereafter, we investigated the partnership between EZH2 and UCA1 to figure out the molecular mechanisms responsible for the regulation of As‐induced cytotoxicity. As shown in **Figure** **3**A, the EZH2 concentration was decreased with a dose‐dependent manner in UCA1‐overexpressed HepG2 cells, indicating a negative regulation of UCA1 on the EZH2 protein. In parallel with the above explorations, we continued to examine the regulation of UCA1 on the expression and degradation of EZH2 protein. Similar to the As‐induced effect, qRT‐PCR analyses demonstrated that the transcriptional levels of EZH2 were not prominently induced by UCA1 in HepG2 cells, compared to the control groups (Figure 3B). As illustrated in Figure 3C, further ubiquitination assays proved that UCA1 overexpression remarkably increased the levels of EZH2 ubiquitination, signifying that UCA1 induced decreased contents of EZH2 by attenuating its protein stability via the ubiquitin–proteasome pathway.

To corroborate the above findings, the concentrations of EZH2 were further analyzed in HepG2 cells incubated with the protein translation inhibitor cycloheximide (CHX). As shown in Figure 3D,E, the cellular EZH2 levels were signally dropped with a time‐dependent property under CHX treatment. Meanwhile, UCA1 overexpression promoted the tendency of ubiquitination and degradation of EZH2, relative to the untreated cells (Figure 3D). Conversely, knockdown of UCA1 attenuated the EZH2 ubiquitination and degradation, compared to the scrambled control cells (Figure 3E). We then detected the role of UCA1 in As‐induced decreased expression of EZH2. As illustrated in Figure 3F,G, the protein levels of EZH2 were ulteriorly reduced with a time‐dependent manner responding to As treatment in the UCA1 overexpression HepG2 cells, and as expected, relieved in the UCA1 knockdown HepG2 cells. Similarly, the regulation of UCA1 in As‐induced decreased expression of EZH2 was testified in multiple cell lines, including human prostate cancer cell line PC3, human breast cancer cell line 231, human lung adenocarcinoma cell line A549, and normal human kidney cell line HK2 (Figure S2, Supporting Information). Therefore, our data again manifested that ubiquitylation and degradation of EZH2 under As treatment were mediated by UCA1.

### 2.4 LncRNA UCA1 Promotes CDK1‐Mediated Degradation of EZH2 Protein

**Figure 4:**
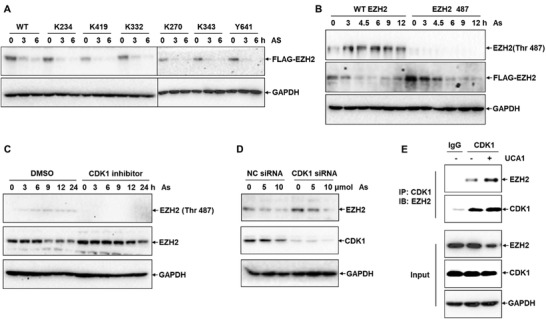
LncRNA UCA1 promotes the degradation of EZH2 via CDK1. A) The degradation of EZH2 in HepG2 cells transfected with FLAG‐EZH2 plasmids including mutated phosphorylation sites (K234, K419, K332, K270, K343, and Y641) in response to 10 µmol As for 0, 3, and 6 h was detected via Western blot analysis (*n* = 3). B) Western blot analysis of the phosphorylation of EZH2 at Thr‐487 site in HepG2 cells exposed to 10 µmol As for 0, 3, 6, 9, 12, and 24 h (*n* = 3). C) The phosphorylation of EZH2 and the protein level of EZH2 in HepG2 cells pretreated with 1 µmol CDK1 inhibitor responding to 10 µmol As at different time points (*n* = 3). D) Western blot analysis of the protein contents of EZH2 and CDK1 in NC siRNA control and CDK1 siRNA cells under indicated dosage of As (*n* = 3). E) Enrichment of EZH2 in HepG2 cells transfected with CDK1 and UCA1 plasmids from CDK1 antibody and normal IgG pulldown complexes was detected by Western blot assay in IP assay (*n* = 3).

It has been well known that the ubiquitination of proteins is relevant to their phosphorylation, and plays a fundamental role in the regulation of protein stability.^[^ ^40^, ^41^ ^]^ Consequently, we set out to seek the key phosphorylation sites responsible for the ubiquitination of EZH2 under As treatment. The EZH2 plasmids with mutated phosphorylation sites, lysine 234 (K234), lysine 419 (K419), lysine 332 (K332), lysine 270 (K270), lysine 343 (K343), tyrosine 641 (Y641), and threonine 487 (Thr 487), were transfected into HepG2 cells to detect the protein stability of these EZH2 mutants under As treatment. As shown in **Figure** **4**A, mutation of the phosphorylation sites at K234, K419, K332, K270, K343, and Y641 did not affect the protein degradation rate of EZH2 under As stress. On the contrary, the protein contents of FLAG‐EZH2 were more stable upon mutating Thr 487 site of EZH2, compared to WT‐EZH2 in response to As treatment (Figure 4B), manifesting that Thr 487 site was responsible for the protein stability of EZH2 in response to As.

Previous studies have reported that kinase CDK1 could induce the phosphorylation of EZH2,^[^ ^42^ ^]^ we thus endeavored to verify the role of CDK1 in the ubiquitination and degradation of EZH2 under As treatment. As illustrated in Figure 4C, the protein levels of EZH2 were progressively decreased in a time‐dependent manner responding to As stress in untreated cells. But in CDK1 inhibitor‐treated cells, EZH2 protein was more stable and only slightly decreased under 24 h As treatment. Likewise, the cellular EZH2 protein was also more stable in CDK1 knockdown HepG2 cells, compared to the scrambled control cells, when in response to As (Figure 4D). Briefly, these data showed that As‐induced EZH2 protein degradation was CDK1 dependent.

Subsequently, we investigated the function of UCA1 in the CDK1‐mediated EZH2 phosphorylation. As shown in Figure 4E, immunoprecipitation (IP) assays proved that overexpression of UCA1 promoted the interaction between CDK1 and EZH2, indicating that UCA1 enhanced the EZH2 phosphorylation induced by CDK1 through promoting their interaction, and further facilitated the degradation of EZH2.

### 2.5 LncRNA UCA1/EZH2 Regulates As‐Induced Cell Cycle Arrest through Activating NFATc2

**Figure 5:**
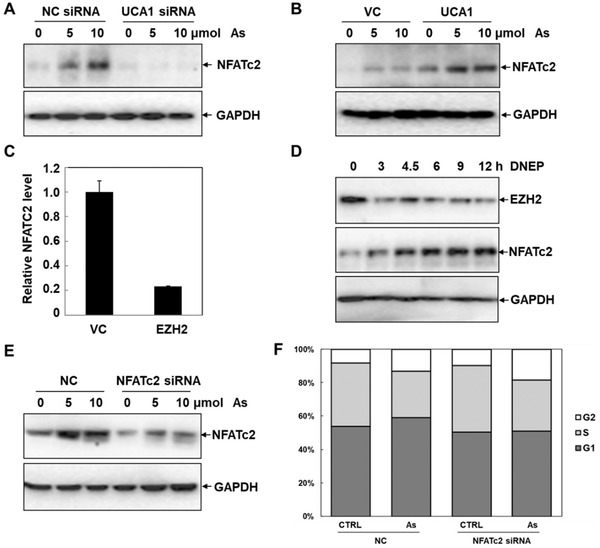
NFATc2 is the downstream target of UCA1/EZH2 complex. A,B) Western blot analysis of NFATc2 protein levels in HepG2 cells transfected with UCA1 siRNA and UCA1 overexpression plasmids in response to indicated concentrations of As (*n* = 3). C) Relative levels of NFATc2 in HepG2 cells transfected with EZH2 plasmids were detected by qRT‐PCR analysis (*n* = 3). D) The protein contents of EZH2 and NFATc2 in HepG2 cells pretreated with 1 µmol DNEP at different time points were analyzed by Western blot analysis (*n* = 3). E) Western blot analysis of the protein contents of NFATc2 in NC siRNA control and NFATc2 siRNA cells exposed to the indicated dosage of As (*n* = 3). F) Cell cycle distribution in scrambled control and NFATc2 siRNA HepG2 cells in response to As was analyzed via flow cytometry, after staining by PI (*n* = 3).

Previous research has indicated that NFAT family members played important roles in the regulation of cell cycle.^[^ ^43^ ^]^ As shown in Figure S3 (Supporting Information), the heatmap showed that the expression of NFATc2 was positively related to the overexpression of UCA1, relative to the control groups, implying that NFATc2 might be the final executor responsible for the As‐induced cell cycle arrest regulated by UCA1/EZH2. Hence, Western blot analyses were carried out to corroborate the regulation of UCA1 on NFATc2 expression. Results revealed that the levels of NFATc2 protein were markedly upregulated with a dose‐dependent property responding to As treatment in both treated and control HepG2 cells. Concretely, knockdown of UCA1 significantly downregulated the expression of NFATc2 (**Figure** **5**A), while overexpression of UCA1 notably enhanced the NFATc2 contents (Figure 5B), demonstrating the positive regulation of UCA1 on NFATc2 protein concentration.

Afterward, we focused on confirming the role of NFATc2 in mediating EZH2‐regulated cell cycle arrest in response to As stress. As illustrated in Figure 5C, qRT‐PCR analyses signified that the transcriptional levels of NFATc2 were prominently decreased with fivefold in EZH2 overexpression HepG2 cells, compared to the control groups. Similarly, the expression levels of NFATc2 were markedly increased in a time‐dependent manner under the treatment of EZH2 inhibitor DNEP (Figure 5D). Next, knockdown of NFATc2 was performed in HepG2 cells by RNAi to explore its regulatory functions in As‐induced cell cycle arrest (Figure 5E). As shown in Figure 5F, flow cytometry analyses illustrated that As‐induced cell cycle arrest was enhanced in the NFATc2 knockdown HepG2 cells, compared to the control cells. These results indicated that NFATc2 was the downstream executor of UCA1/EZH2 signaling, which was activated to antagonize As‐induced cell cycle arrest.

## 3 Discussion

As is a recognized toxic metalloid element widely distributed in the environment, and results in a series of chronic or acute human health issues.^[^ ^1^, ^7^, ^44^ ^]^ Till now, existing evidence suggested that cell cycle arrest is the adverse biological effect responding to As, and correlative with various toxic effects and complications, including carcinogenesis.^[^ ^7^, ^45^, ^46^ ^]^ Whereas, the underlying epigenetic molecular mechanisms of cell cycle arrest remain elusive, especially in the case of environmental contamination exposure. In this article, more hepatocytes arrested in the G2/M stage were observed in the As‐treated groups through flow cytometry, which was in accord with the previous studies.^[^ ^38^ ^]^ Our combined results unraveled that lncRNA UCA1, protein EZH2, and NFATc2 participated in the regulation of cell cycle arrest, which contributed to exploring the molecular mechanisms responsible for As toxicity. Since the regular cell cycle plays a crucial role in cells’ survival and organism's health,^[^ ^47^, ^48^ ^]^ our research identified As exposure as the causative factor for cell cycle arrest.

The methyltransferase EZH2 could catalyze H3K27me3 to promote the epigenetic silencing, and has been considered as an essential regulator in various cellular progresses, tumorigenesis, and organism homeostasis.^[^ ^49^, ^50^, ^51^ ^]^ Moreover, convincing studies have testified that EZH2 also interacts with lncRNAs to further regulate the expression of downstream genes, which plays important roles in the epigenetic regulation.^[^ ^52^, ^53^ ^]^ In the current work, we focused on confirming the interaction between UCA1 and EZH2 on the structural level, and the phosphorylation of EZH2 induced by kinase CDK1 at Thr‐487 site. Further mechanistic investigations manifested that UCA1 could bind to EZH2 to enhance its phosphorylation through recruiting CDK1, which facilitated EZH2 ubiquitination and degradation. On the other hand, our findings indicated that As exposure potentiated the degradation of EZH2 after its ubiquitination. Linking to that EZH2 knockdown attenuated the As‐induced cell cycle arrest, which implied a potential prosurvival signaling pathway to antagonize As‐induced cytotoxicity.

It is well accepted that lncRNA UCA1 is a crucial epigenetic regulator in the pathogenesis and progression of cancer.^[^ ^29^ ^]^ A growing number of studies have verified the overexpression of UCA1 in carcinoma tissues compared with the adjacent normal tissues, such as hepatocellular carcinoma,^[^ ^54^ ^]^ bladder cancer,^[^ ^55^ ^]^ and breast cancer.^[^ ^56^ ^]^ Besides, UCA1 has been reported to be correlated with tumor proliferation and metastasis.^[^ ^57^ ^]^ For example, the silence of UCA1 in hepatoma cells inhibited tumor growth in nude mice.^[^ ^54^ ^]^ The oncogenic effect of UCA1 is derived from its promoting role in cell proliferation and cycle progression.^[^ ^58^ ^]^ Therefore, UCA1 is characterized as a diagnostic and predictive biomarker for human cancer.^[^ ^59^, ^60^ ^]^ Nevertheless, the underlying function and mechanism of UCA1 in the complex pollutant‐associated signaling network remain unclear. Our previous study has uncovered that UCA1 was induced to inhibit autophagy‐dependent cell death upon As stress.^[^ ^32^ ^]^ Here, we provided evidence uncovering the prosurvival function of UCA1, which attenuated As‐induced cell cycle arrest through binding to EZH2 and enhancing the latter's phosphorylation and ubiquitination.

Cell cycle progression plays pivotal roles in the normal growth and development of cells, tissues and organisms, consists of four phases, including the G0/G1, S, G2, and M phases.^[^ ^61^, ^62^ ^]^ Cell cycle arrest is the phenomenon that cell division is blocked in G1 or G2 phases, which is under the control of cyclins and cyclin‐dependent kinases (CDKs).^[^ ^63^ ^]^ Under this context, cell cycle arrest is considered as a protection mechanism to avoid anomalous hereditary information entering the daughter cells.^[^ ^64^ ^]^ Several studies have revealed that contaminant exposure could induce cell cycle arrest in numerous cell types.^[^ ^65^, ^66^, ^67^ ^]^ For instance, the cooking oil fume derived PM2.5 was capable of causing G0/G1 cell arrest in AEC II pneumocytes.^[^ ^68^ ^]^ Cr(VI) exposure resulted in the G2/M phase arrest through ROS in L‐02 hepatocytes.^[^ ^69^ ^]^ Given the regular progression and precise regulation of cell cycle are critical to the cells and organisms,^[^ ^70^ ^]^ cell cycle arrest, especially G2/M arrest,^[^ ^71^ ^]^ would trigger manifold problems, including cell death.^[^ ^38^ ^]^ To date, the underlying molecular mechanisms of contaminant‐induced cell cycle deregulation have not been characterized in detail. NFAT family consists of five different isoforms, including NFATc1, NFATc2, NFATc3, NFATc4, and NFAT5. NFATs have been documented to participate in the regulation of multiple biological processes, such as cell cycle, cell differentiation and so on.^[^ ^72^, ^73^ ^]^ Importantly, NFAT signaling activation could induce cell cycle transition via influencing the expression of cyclin, suggesting a vital involvement of NFAT family in the cell death and tumorigenesis.^[^ ^43^, ^74^, ^75^ ^]^ In fact, this is the first report uncovering that NFATc2 is the pivotal executor in the regulation of As‐induced cell cycle arrest. In the current study, we demonstrated that As‐induced cell cycle arrest was negatively regulated by NFATc2. Mechanistically, the degradation of ubiquitination‐regulated EZH2 promoted the increase of NFATc2 expression, and further antagonized the As‐blocked cell cycle. Nevertheless, our findings are still a tip of the iceberg concerning the research of As‐induced toxicity, and the intensive investigation is deserved in the future.

**Figure 6:**
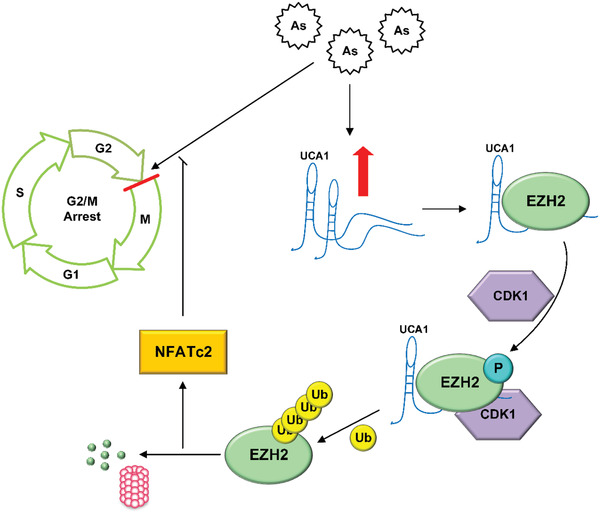
A schematic depicting the interplay of UCA1 with EZH2 to mediate As‐induced G2/M phase arrest. Interaction between UCA1 and EZH2 protein resulted in latter phosphorylation and ubiquitylation mediated by CDK1, consequently activating NFATc2 to antagonize the As‐blocked cell cycle.

To summarize, this current study unearths a novel prosurvival signaling pathway conducted by EZH2 under As‐induced cell cycle arrest. As illustrated in **Figure** **6**, As‐responsive lncRNA UCA1 interacts with EZH2 to potentiate its phosphorylation mediated by CDK1, and in turn enhances the ubiquitylation of EZH2 through ubiquitin–proteasome pathway. As a consequence of EZH2 degradation, NFATc2 is activated and further acts to antagonize the As‐blocked cell cycle. Our present study obtains novel insights into the complex networks of defense mechanisms against As threat, and opens a new path to understand the deregulation of cell cycle progression under pollutant exposure.

## 4 Experimental Section

##### Cell Culture

Human liver hepatocellular cell line HepG2 was obtained from the Cell Resource Center of the Institute of Basic Medical Sciences (CAMS, China). Cells were cultivated in the Dulbecco's modified Eagle's medium (Hyclone, USA) containing 10% fetal bovine serum (FBS) (Hyclone), 100 IU mL^−1^ penicillin and 100 µg mL^−1^ streptomycin, in the cell incubator with 5% CO_2_ at 37 °C.

##### Cell Transfection

The small interfering RNA (siRNA) oligos of EZH2, UCA1, CDK1, NFATc2, and negative control siRNA were obtained from GenePharma Biotechnology (Shanghai, China). HepG2 cells were transfected with 2 µg overexpression plasmids of UCA1 and EZH2 or 20 µmol siRNAs using lipofectamine 2000 (Invitrogen) following the instructions of manufacturer.

##### FISH Assays

FISH assays were performed via fluorescent in situ hybridization kit (RiboBio, China) following the protocols of manufacturer. Briefly, HepG2 cells on the slides were fixed in 4% formaldehyde and pretreated with protease K. After dehydrated via anhydrous ethanol, the dried cells were incubated with the hybridization reaction solution, and decontaminated with a series of washing processes. Finally, staining the slides with DAPI and detecting the fluorescent signals by the confocal laser scanning microscope.

##### Cell Cycle Distribution Analysis

Cells were collected and resuspended in PBS at the concentration of about 1 × 10^6^ cells mL^−1^. After fixed in the precooled 70% ethanol, cells were stained by propidium iodide (PI) following the instructions of manufacturer (BD Biosciences, USA). The cell cycle distribution was analyzed via flow cytometry, which divided into G0/G1, S, G2, and M phases according to the disparate DNA contents.

##### IP Analysis

Ubiquitination was detected through IP analysis. Briefly, HepG2 cells were harvested and lysed in the IP lysis buffer after transfected with the plasmids of UCA1 and HA‐tagged ubiquitin. The magnetic beads conjugated with indicated antibodies were formed to immunoprecipitate proteins in the supernatant from cell lysates. After purified with series of washing processes, the precipitated proteins were isolated by sodium dodecyl sulfate‐polyacrylamide gel electrophoresis (SDS‐PAGE), and then analyzed by Western blotting.

##### Western Blot Analysis

Cells were collected and lysed by RIPA buffer (Solarbio, Beijing, China) supplemented with protease inhibitors (Roche, Switzerland). After measured the protein contents via Brandford assays Kit (Solarbio, Beijing, China), total proteins were resolved through SDS‐PAGE, and then immunoblotted and visualized by Western blotting.

##### Real‐time qRT‐PCR Analysis

Total RNAs were extracted from differently treated cells by TRIzol reagent (Invitrogen, USA) according to the instructions of manufacturer. After the quantitative analyses using spectrophotometer, 2–5 µg RNAs were reverse transcribed into cDNAs by RevertAid First Strand cDNA Synthesis Kit (Thermo Fisher Scientific, USA). Thereafter, cDNAs were analyzed through the iQ5 qRT‐PCR instrument (Bio‐Rad). The primer sequences for this study are listed in Table S1 (Supporting Information).

##### Statistical Analysis

All quantitative data were shown as the means ± standard deviation (SD). Statistical analysis was carried out with the independent *t*‐test or one‐way ANOVA test. Experimental data were analyzed by SPSS software; for all analyses, *p*‐value less than 0.05 (*) or 0.001 (#) were identified as significant differences.

## Conflict of Interest

The authors declare no conflict of interest.

## Supporting information

Supporting Information
