# Supplementary material for: LncRNA UCA1 Antagonizes Arsenic‐Induced Cell Cycle Arrest through Destabilizing EZH2 and Facilitating NFATc2 Expression
Source: Adv Sci (Weinh). 2020 Apr 13;7(11):1903630. doi: 10.1002/advs.201903630 (PMC7284218; doi:10.1002/advs.201903630)
Supplement: Supplementary file 1 — Supporting Information [file ADVS-7-1903630-s001.pdf]

LncRNA UCA1 antagonizes arsenic-induced cell cycle arrest through destabilizing EZH2 and facilitating NFATc2 expression

Zheng Dong[#], *Ming Gao*[#] *, *Changying Li, Ming Xu, Sijin Liu**

Z. Dong, M. Gao, M. Xu, Pro. S. Liu

State Key Laboratory of Environmental Chemistry and Ecotoxicology

Research Center for Eco-Environmental Sciences

Chinese Academy of Sciences

Beijing 100085, China

E-mail: sjliu@rcees.ac.cn; minggao@rcees.ac.cn

Z. Dong, M. Gao, M. Xu, Pro. S. Liu

University of Chinese Academy of Sciences

Beijing 100049, China

C. Li

Liver Research Center, Beijing Friendship Hospital

Capital Medical University

Beijing 100050, China.

# These authors equally contribute to this work.

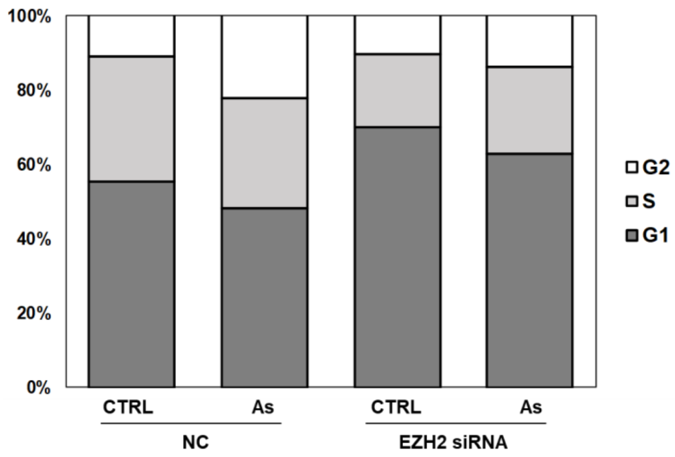

Figure S1. EZH2 regulates As-induced cell G2/M phase arrest in HK2 cells. Flow cytometry analyses determined the cell cycle distribution in normal human kidney HK2 cells transfected with scrambled control and EZH2 siRNA responding to AS, after staining by IP (n=3).

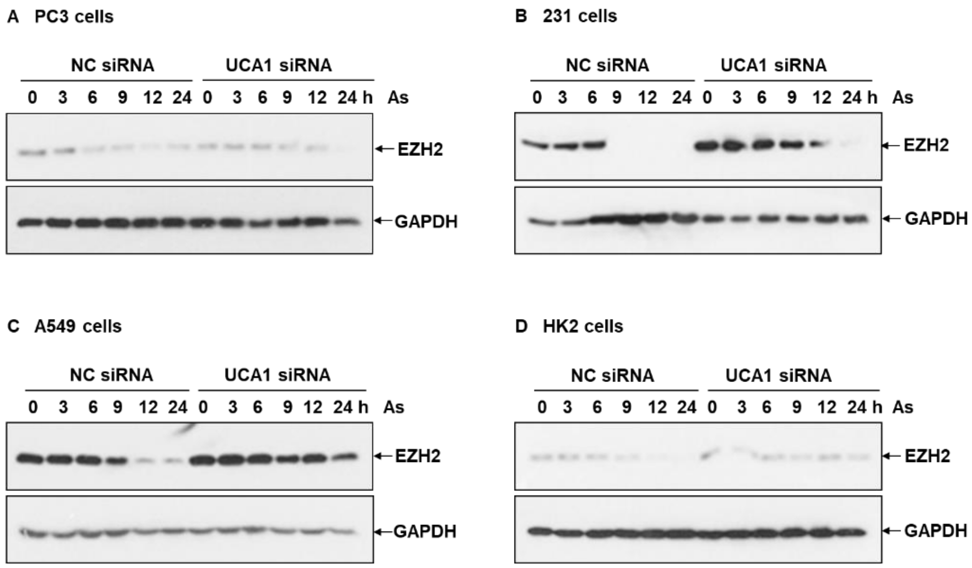

Figure S2. LncRNA UCA1 regulates As-mediated EZH2 expression in different cell lines. Western blot analysis of the expression of EZH2 in PC3 (A), 231 (B), A549 (C) and HK2 (D) cells transfected with UCA1 siRNA exposed to 10 μmol As at different time points (n=3).

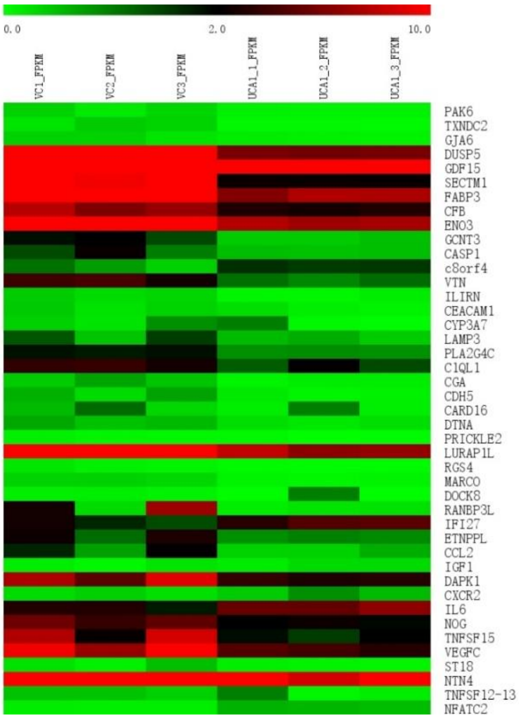

Figure S3. Heat map of gene expression under the overexpression of UCA1 in HepG2 cells. Heat map of these dysregulated genes was built by using GenePattern software.

Table S1 Primers sequences used in this study.

Genes	Sequences (5'-3')
EZH2 F	AATCAGAGTACATGCGACTGAGA
EZH2 R	GCTGTATCCTTCGCTGTTTCC
lncRNA UCA1 F	TTTGCCAGCCTCAGCTTAAT
lncRNA UCA1 R	TTGTCCCCATTTTCCATCAT
CDK1 F	AAGCTGGCTCTTGGAAATTGA
CDK1 R	ATGGCTACCACTTGACCTGTAGTT
GAPDH F	GAAGGTGAAGGTCGGAGT
GAPDH R	GAAGATGGTGATGGGATTTC
NFATc2 F	GAGGGGCTGTCAAAGCTCC
NFATc2 R	ACAGTTTTCCCCGTGATTCGG
